# Examining Anxiety and Insomnia in Internship Students and Their Association with Internet Gaming Disorder

**DOI:** 10.3390/jcm13144054

**Published:** 2024-07-11

**Authors:** Tahani K. Alshammari, Aleksandra M. Rogowska, Anan M. Alobaid, Noor W. Alharthi, Awatif B. Albaker, Musaad A. Alshammari

**Affiliations:** 1 Department of Pharmacology and Toxicology, College of Pharmacy, King Saud University, Riyadh 11451, Saudi Arabia; abaker@ksu.edu.sa (A.B.A.); malshammari@ksu.edu.sa (M.A.A.); 2 Institute of Psychology, University of Opole, 45-052 Opole, Poland; 3 College of Pharmacy, King Saud University, Riyadh 11451, Saudi Arabia; 438202720@student.ksu.edu.sa (A.M.A.); 438201681@student.ksu.edu.sa (N.W.A.)

**Keywords:** insomnia, anxiety, gaming disorder, intern students, mental health, network analysis

## Abstract

**Background:** Internships are a mandatory graduation requirement to help medical students transition to the work environment. Some individuals are prone to anxiety in an unfamiliar environment, which is a public concern among young adults. Here, we investigated the mechanism between internet gaming disorder and anxiety and insomnia among internship students. **Methods:** A convenient sample of 267 internship students was collected in a cross-sectional study module between 17 July and 27 December 2022. The survey contained a 7-item Generalized Anxiety Disorder (GAD-7), Athens Insomnia Scale (AIS), and Internet Gaming Disorder Scale—Short-Form (IGDS9-SF). The association was estimated using Pearson’s correlations, and network analysis was performed to characterize these associations. **Results:** Our results indicate that about 60% of participants exhibited mild to severe anxiety and insomnia, while 2.28% showed symptoms of internet gaming disorder. Also, we found a moderate association between anxiety and insomnia. An item-level analysis indicated that GAD_1 “feeling anxious” and GAD_5 “unable to sit still” are essential for gaming, and that GAD_2 “uncontrollable worrying” is crucial for insomnia. This indicated an interplay between these items, supported by our centrality analysis, where we found that GAD_1 and GAD_2 depicted high centrality. **Conclusions:** We found high rates of anxiety and insomnia in internship students and the association between selected symptoms of anxiety and insomnia. At the same time, low rates of internet gaming disorder could be attributed to a lack of time for entertainment and an increased awareness of its risks. Given these findings, an awareness of anxiety and insomnia risk should be emphasized.

## 1. Introduction

Mental health disorders such as anxiety, depression, sleep difficulty, and addiction behavior are serious psychological and health problems. People living under stress, such as undergraduates and interns, are more subjected to such conditions [1]. Reports indicated a higher risk of developing anxiety and depression in undergraduate students in the first and second waves of the pandemic [2,3]. Further, the prevalence of depression during the second wave of COVID-19 was documented as high as 50% in cross-sectional settings [4], indicating the rate of psychological stress has been elevated since the pandemic. In line with this, a two-year longitudinal post-COVID-19 pandemic study examined the trajectories of mental health aspects in the Italian population. The study indicated a reduction in depression, anxiety, and sleep disturbances. However, the duration of evening sleep was gradually reduced in young individuals [5].

The internship is a transition phase where students acquire practical experience and become familiar with the work environment [6]. Internships during the final undergraduate year strongly dictate the professional transition to practice and employment [7]. Interns had a higher prevalence of psychological distress when compared to the general population [8]. Psychological conditions could cause changes in behaviors and personality. These changes could be driven by the long working hours associated with continuous sleep deprivation [9]. Other challenges interns face are adjustment issues and building their professional competence, which includes interpersonal and communication skills, knowledge, experience, adapting to a decision-making role, difficulty with the licensure exam, and job pursuit [8,10]. 

Online gaming has become a public health concern among young adults, as it can lead to addiction. Excessive online gaming is becoming a behavior pattern that causes harmful consequences at physiological and psychological levels. This behavior can be severe enough to damage personal, family, social, educational, occupational, and other critical aspects of functioning [11]. As various studies show a high prevalence of anxiety and insomnia among intern students [12,13,14], it is essential to understand the particular pattern of anxiety and insomnia associations in relation to online gaming.

Insomnia is a common sleep disorder characterized by difficulty falling or staying asleep, usually accompanied by daytime impairment [4]. Chronic sleep restriction is linked to daytime cognitive deficit, metabolic dysfunction, and alterations in the endocrine system [15]. Further, insomnia is related to somatization and emotional dysregulation [16]. Also, a volumetric analysis of the brains of patients with anxiety disorder showed abnormalities in magnetic resonance readout, and these alterations were associated with the severity of insomnia [17].

It has been reported that insomnia is higher among older people, females, and people with medical and psychiatric conditions [4]. However, research studies and reviews have highlighted the prevalence of insomnia and sleep deprivation in residents and interns [12,18,19,20]. In line with this, a survey-based report showed that short sleep duration was associated with anxiety during the internship, and predicted future anxiety symptoms [12]. 

We previously reported, using regression and mediation models, that anxiety and insomnia mediate gaming addiction and depression association [21]. In another report, we found that health sciences college students exhibited a higher level of anxiety; more than 50% of the participants reported moderate to severe anxiety [22]. Further, in the second wave of COVID-19, we found a significant number of college students were at risk of anxiety and depression, and there was an association between these risks and poor sleep quality [23]. We further examined the risks of insomnia in Saudi students, and more than 70% of the participants were at higher risk of experiencing insomnia while addressing the Athens Insomnia Scale [24].

A two-year longitudinal study indicated more than a third of college students exhibited insomnia, and those with psychological determinants, including perfectionism and low self-esteem, were predisposed to it [25]. In line with this, another report examining the mental well-being of undergraduate nursing students indicated that more than 70% of the participants exhibited higher risks of insomnia [26]. Also, a study conducted at Bielefeld University reported that more than 70% of the study participants presented insomnia symptoms [27]. Further, students with insomnia reported clinical and non-clinical conditions, including anxiety, depression, stress, and fatigue [28,29,30]. Thus, the relationship between insomnia and mental health issues is well acknowledged. Further, insomnia is associated with addiction [31,32] and alcohol use [33,34], indicating an intervention is paramount at the organizational level to raise awareness about the clinical consequences of insomnia. 

Reports have indicated that the severity of internet gaming disorder is linked to anxiety and poor sleep quality [35,36,37]. Fazeli et al. [38] found a mechanistic indication that anxiety is a significant mediator of insomnia and internet gaming disorder. Yet, these reports were based on total scores, which makes it difficult to intervene and target a specific symptom.

Network analysis is an emerging tool for understanding the complex relations of mental disorders’ comorbidities. The structural foundation is a dimensional module composed of nodes and edges. Nodes represent symptoms, while edges reflect the relationship between these nodes. This analysis module would determine the most influential–central–symptom, identifying an appropriate symptom-based intervention [39,40]. 

In the context of psychiatric disorders, network analysis has been utilized to delineate the comorbid symptoms of anxiety and depression [41,42,43,44], depression and psychological factors [45,46], eating disorders, and psychiatric diseases [47]. Yet, most of these reports were conducted during the COVID-19 pandemic. Additionally, only a few studies examined insomnia and anxiety. However, these studies included other conditions and factors, such as depression and workload [48,49,50]. The existence of additional multivariates could add further heterogeneity to the insomnia–anxiety network analysis [39]. 

In a clinical setting, anxiety and insomnia are linked to economic, social, and cultural components [51,52,53,54,55]. Thus, findings from studies conducted in Africa, North and South Asia, and Western countries might not be applicable. Even though interns face challenges of novel and stressful training environments along with maintaining sufficient sleep hours, a comprehensive study examining this sample population has not been conducted. As far as we know, no studies based on network analysis at an item-level analysis examined the anxiety–insomnia–gaming network in the internship sample population. Also, none have been established in Middle Eastern countries. Here, we aimed to explore the complex relationships between the symptoms of insomnia, anxiety, and internet gaming disorder among internship students using network analysis. 

## 2. Materials and Methods

A cross-sectional study was conducted on male and female internship-year students from King Saud University from 17 July to 27 December 2022. An online survey was generated by Google Forms in Arabic and English and distributed to participants using convenient sampling technology through Twitter, WhatsApp, and Telegram. An electronic consent notification, indicating that the participant agrees to participate in the study and that the response is entirely confidential and voluntary, along with a clear indication of the objectives and descriptions of the project, was shown at the beginning of the survey. Ethical approval was obtained from the Institutional Review Board at King Saud University (KSU), Riyadh, Saudi Arabia (Ref No. 22/0055/IRB), approved on the 23rd of January 2022. Although 267 students responded to invitations, four were excluded because of missing data, totaling more than 5%. Finally, the data of 263 participants were statistically analyzed. 

### 2.1. Participants

A total of 263 students, aged between 18 and 30 (*M* = 22.83, *SD* = 1.37), participated in the study, including 67.68% (*n* = 178) of females and 32.32% (*n* = 85) of males, 95.82% (*n* = 252) of singles and 4.18% (*n* = 11) of married (Table 1). The number of family members at home ranged between 1 and 14, with a mean of 6 (*M* = 6.41, *SD* = 2.35). The participants in the study were interns who were studying at Saudi institutes from different colleges, which include the college of pharmacy (*n* = 55, 20.91%), college of dentistry (*n* = 18, 6.84%), college of medicine (*n* = 10, 3.80%), college of nursing (*n* = 41, 15.59%), college of applied medical sciences (*n* = 120, 45.63%), and college of humanities (*n* = 2, 0.76%).

### 2.2. Measurements

#### 2.2.1. Anxiety

The Generalized Anxiety Disorder (GAD-7) scale is a validated 7-item used widely and easily to screen for anxiety in general and research settings [56]. The GAD-7 scale uses a four-point Likert scale from 0 (not at all) to 3 (every day), with total scores ranging from 0 to 21; higher scores represent higher grades of anxiety. The total scores of 5, 10, and 15 are taken as the cut-off points for mild, moderate, and severe anxiety, respectively. Both English, Cronbach’s alpha = 0.92 [57], and Arabic, Cronbach’s alpha = 0.95 [58] versions of this tool were used in the study.

#### 2.2.2. Insomnia 

The Athens Insomnia Scale (AIS) is an 8-item questionnaire developed to assess insomnia, demonstrating strong consistency, reliability, and validity [59]. The first five items assess problems with sleep induction, awakening during the night, early morning awakening, total sleep duration, and overall sleep quality. The last three items are about the next-day consequences of insomnia, such as problems with a sense of well-being, functioning, and sleepiness during the day. Each AIS item is scored on a 0-to-3 scale, with 0 representing no problem and 3 representing a profoundly serious problem. The overall score on these eight items varied from 0 to 24, with a score of AIS ≥ 6 indicating insomnia. Subjects were asked to rate positively if they had experienced the item at least thrice weekly in the previous month. Both English, Cronbach’s alpha = 0.84 [60], and Arabic, Cronbach’s alpha = 0.83 [61] versions of this tool were used in the study.

#### 2.2.3. Gaming

The Internet Gaming Disorder Scale 9—Short-Form (IGDS9-SF) is the most commonly used psychometric tool for evaluating the severity of an internet gaming disorder over a year [62,63]. The IGDS9-SF incorporates all nine internet gaming disorder criteria established by the American Psychiatric Association (APA) in the DSM-5, with attributes of conciseness and short administration time, making it useful in clinical and research settings. Furthermore, the IGDS9-SF’s psychometric properties, including internal consistency and validity, have been thoroughly evaluated [64]. The scale uses a 5-point Likert-type: Never (1), Rarely (2), Sometimes (3), Often (4), and Very Often (5). The total score is calculated by summing the nine items (range: 9–45), with a higher score indicating a higher level of internet gaming disorder (IGD). A response of “Very often” on five or more of the nine items on the IGDS9-SF is considered indicative of IGD [65]. Both English, Cronbach’s alpha = 0.91 [65], and Arabic, Cronbach’s alpha = 0.92 [66] versions of this tool were used in the study.

### 2.3. Demographics

The demographic characteristics assessed in the study are participants’ age, gender, marital status, number of family members at home, and internship program.

### 2.4. Statistical Analysis

Descriptive statistics were performed to examine the parametric properties of variables, including range of scores, mean (*M*), standard deviation (*SD*), skewness, and kurtosis. Reliability was assessed using Cronbach’s α coefficients. Pearson’s correlation analysis was conducted to examine associations between internet gaming disorder, anxiety, and insomnia symptoms. Finally, the network analysis (NA) was implemented to identify the most important variables for the model of associations between particular symptoms of insomnia, general anxiety disorder, and internet gaming disorder among internship students. We used the NA with extended Bayesian information criteria and graphical least absolute shrinkage, and selection operator (EBICglasso) as an estimator. The weighted network between nodes is represented by magnitude (the thicker the lines between nodes, the stronger the relationship). The closeness between nodes also shows the strength of correlations. Closeness centrality quantifies how close a node is to all other nodes in the network. It is calculated as the inverse of the sum of the shortest path distances between a node and all other nodes in the network. Several centrality indices (i.e., betweenness, closeness, degree, and expected influence) identified the network model’s most relevant, influential, and crucial variables. All statistical tests were performed using the JASP ver. 0.16.1.0. software for Windows.

## 3. Results

### 3.1. Prevalence of Internet Gaming Disorder, Anxiety, and Insomnia Symptoms

About 87 of our participants responded to the Arabic questionnaire, and 167 responded to the English version. The frequencies of particular categories of disordered symptoms are shown in Table 1. Among participants, only six (2.28%) students met the criteria of gaming addiction (i.e., a minimum of 5 out of 9 items of the IGDS9-SF rated as “very often”). Moderate or severe symptoms of general anxiety disorder (GAD-7 ≥ 10) were found in 28.90% of participants (see Table 2 for more details). Most of the interns (60%) presented insomnia symptoms (AIS ≥ 6). 

### 3.2. Associations between Internet Gaming Disorder, Anxiety, and Insomnia Symptoms

Descriptive statistics are presented in Table 3. Since the sample size was quite large (*N* > 200), and skewness and kurtosis ranged between +0.50, the properties of internet gaming disorder, general anxiety disorder, and insomnia symptoms were appropriate for further parametric statistical tests.

**Figure 1:**
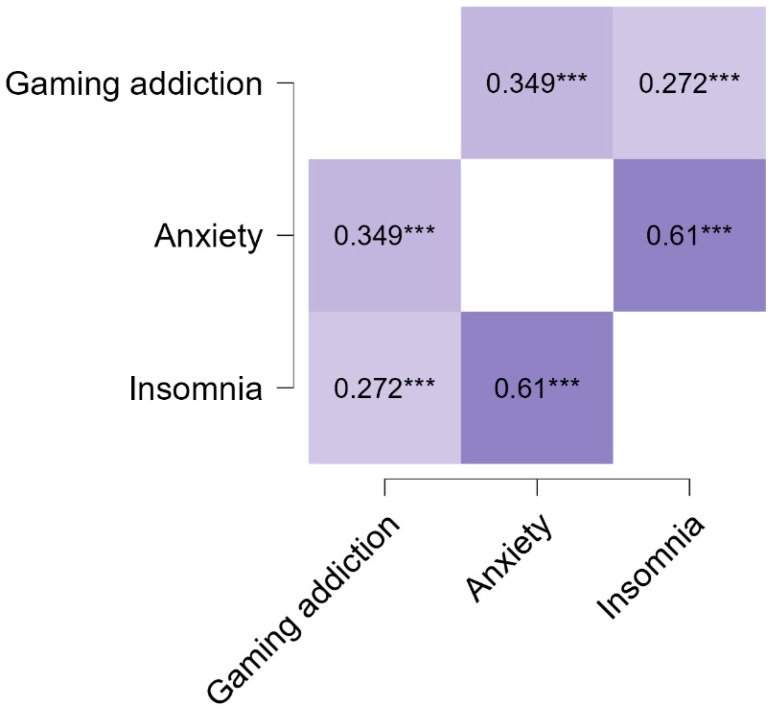
Pearson’s correlations between internet gaming disorder, anxiety, and insomnia symptoms (*N* = 263). *** *p* < 0.001.

A Pearson’s correlation analysis showed that internet gaming disorder is related to anxiety symptoms, *r* = 0.35, [95% CI (0.24, 0.45)], *p* < 0.001 (small strength). Insomnia symptoms are linked to internet gaming disorder, *r* = 0.27, [95% CI (0.16, 0.38)], *p* < 0.001 (small strength). Also, a moderate association was found between general anxiety disorder and insomnia symptoms, *r* = 0.61, [95% CI (0.50, 0.70)], *p* < 0.001. All associations were positive (see Figure 1).

### 3.3. Network Analysis

**Figure 2:**
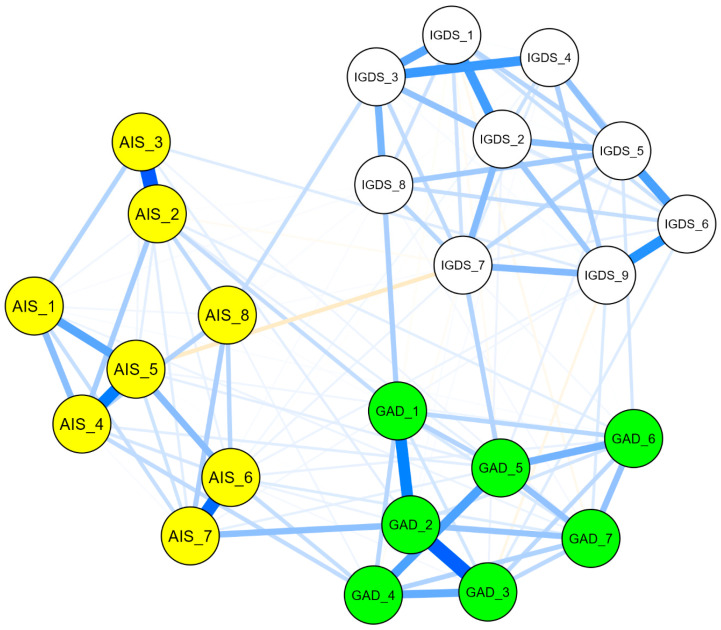
Network structure of associations between the insomnia (AIS), anxiety (GAD), and gaming disorder (IGDS) scales. Note. The blue line represents a positive relationship, while the orange line indicates a negative association between nodes in the network model. AIS = Athens Insomnia Scale, AIS_1 = delaying sleep, AIS_2 = problems with waking during the night, AIS_3 = final awakening earlier than desired, AIS_4 = sense of total sleep duration sufficiency, AIS_5 = overall satisfaction of sleep quality, AIS_6 = sense of well-being during the day, AIS_7 = physical and mental functioning during the day, AIS_8 = sleepiness during the day. GAD = General Anxiety Disorder, GAD_1 = feeling nervous, anxious, or on edge, GAD_2 = not being able to stop or control worrying, GAD_3 = worrying too much about different things, GAD_4 = trouble relaxing, GAD_5 = being so restless that it is hard to sit still, GAD_6 = becoming easily annoyed or irritable, GAD_7 = feeling afraid as if something awful might happen. IGDS = Internet Gaming Disorder Scale, IGDS_1 = preoccupation with online/offline gaming, IGDS_2 = experience of unpleasant symptoms when gaming is taken away, IGDS_3 = the need to spend increasing amounts of time engaged in games, IGDS_4 = unsuccessful attempts to control participation in games, IGDS_5 = loss of interest in previous hobbies and entertainment as a result of, and with the exception of, games, IGDS_6 = continued excessive use of games despite knowledge of psychosocial problems, IGDS_7 = deceiving family members, therapists, or others regarding the amount of gaming, IGDS_8 = use of games to escape or relieve negative moods, IGDS_9 = jeopardizing or losing a significant relationship, job, or education or career opportunity because of participation in games.

The network analysis was performed to examine the complex relationships between particular symptoms of insomnia, anxiety, and internet gaming disorder among internship students. The structure of associations is presented in Figure 2. Our findings indicated a positive relationship association, and they indicated that a peripheral cluster existed within the internet gaming disorder scale. It is composed of IGDS_1, “the preoccupation with online/offline gaming” IGDS_3, “the need to spend increasing amounts of time engaged in games” IGDS_4, “unsuccessful attempts to control participation in games”, and the IGDS_2, “experience of unpleasant symptoms when gaming is taken away” nodes.

Another connection was detected between IGDS_5, “loss of interest in previous hobbies”, IGDS_6, “continued excessive use of games despite knowledge of psychosocial problems”, and IGDS_9, “jeopardizing or losing a significant relationship, job, or education opportunity because of participation in games”, nodes. A moderate association was observed between IGDS_3, “the need to spend increasing amounts of time engaged in the games” node, and IGDS_8, “the node of use of games to escape or relieve negative moods”.

The nodes GAD_1, “feeling nervous”, and GAD_2, “uncontrollable worrying”, were strongly connected. The nodes GAD_3, “worrying too much”, and GAD_4, “ trouble relaxing”, were strongly clustered. A direct association was observed between GAD_5 “restlessness” and GAD_6 “irritability” nodes.

The insomnia network indicated a strong association between AIS_2 “awakening problems” and AIS_3 “awakening earlier than desired” nodes. Similarly, the nodes of AIS_4, “sense of sleep duration sufficiency”, and AIS_5, “satisfaction of sleep quality”, were interconnected. Additionally, the nodes of AIS_6, “sense of well-being during the day”, and AIS_7, “physical and mental functioning during the day”, were strongly connected.

With the IGDS-GAD model, IGDS_8, “the use of games to escape or relieve negative moods”, and GAD_1, “the feeling nervous” nodes, had the strongest connection. Another direct positive connection was detected between GAD_5, “the restlessness” node, and IGDS_7, “the deceiving family members or others regarding the amount of gaming” node.

With the AIS-GAD model, the nodes of AIS_6 “sense of well-being during the day”, and AIS_4 “sense of sleep duration sufficiency” were directly connected to GAD_4 “trouble relaxing”. Also, the node of AIS_7 “physical and mental functioning during the day” was directly connected to the GAD_2 node. Another direct positive connection was detected between the GAD_1 “feeling nervous” and AIS_2 “awakening problems” nodes.

With the AIS-IGDS model, a negative association was detected between the AIS_5 “satisfaction of sleep quality” node and IGDS_7 “deceiving family members or others regarding the amount of gaming” node.

**Figure 3:**
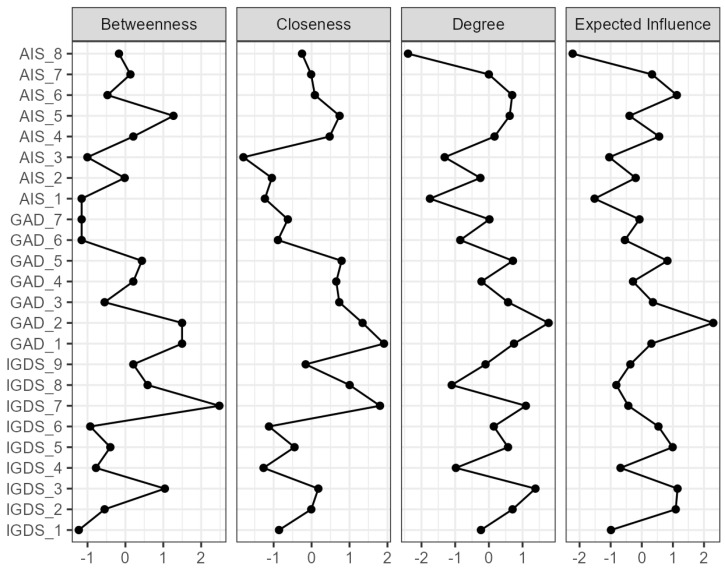
Centrality plot. Note. AIS = Athens Insomnia Scale, AIS_1 = delaying sleep, AIS_2 = problems with waking during the night, AIS_3 = final awakening earlier than desired, AIS_4 = sense of total sleep duration sufficiency, AIS_5 = overall satisfaction of sleep quality, AIS_6 = sense of well-being during the day, AIS_7 = physical and mental functioning during the day, AIS_8 = sleepiness during the day. GAD = General Anxiety Disorder, GAD_1 = feeling nervous, anxious, or on edge, GAD_2 = not being able to stop or control worrying, GAD_3 = worrying too much about different things, GAD_4 = trouble relaxing, GAD_5 = being so restless that it is hard to sit still, GAD_6 = becoming easily annoyed or irritable, GAD_7 = feeling afraid as if something awful might happen. IGDS = Internet Gaming Disorder Scale, IGDS_1 = preoccupation with online/offline gaming, IGDS_2 = experience of unpleasant symptoms when gaming is taken away, IGDS_3 = the need to spend increasing amounts of time engaged in games, IGDS_4 = unsuccessful attempts to control participation in games, IGDS_5 = loss of interest in previous hobbies and entertainment as a result of, and with the exception of, games, IGDS_6 = continued excessive use of games despite knowledge of psychosocial problems, IGDS_7 = deceiving family members, therapists, or others regarding the amount of gaming, IGDS_8 = use of games to escape or relieve negative moods, IGDS_9 = jeopardizing or losing a significant relationship, job, or education or career opportunity because of participation in games.

Figure 3 depicts the centrality plot, and Supplementary Table S1 exhibits the centrality analysis values. Our analysis indicated that IGDS_7, “deceiving family members or others regarding the amount of gaming”, is more critical for betweenness centrality. Nodes with high closeness centrality can quickly interact with other nodes in the network. In the present study, IGDS_7 and GAD_1 are critical for closeness centrality. In addition, GAD_2 determines the degree and expected influence of centrality.

## 4. Discussion

Network analysis is a novel approach to understand the complex interactions between symptoms and conditions. In the context of the anxiety–insomnia–gaming network, the analysis can identify which symptoms are most strongly interconnected and how they may be contributing to each other’s severity. In this work, we found elevated rates of anxiety and insomnia in internship students. A small percentage of our study participants exhibited gaming addiction. Further, we identified an association between selected symptoms of the anxiety–insomnia–gaming network. The item-level analysis indicated that GAD_1, “feeling anxious”, and GAD_5, “restlessness”, are central to gaming and that GAD_2, “uncontrollable worrying”, is central to insomnia. This indicates an interplay between these items, supported by our centrality analysis, where we found that GAD_1 and GAD_2 depicted high centrality.

The rates of anxiety and insomnia are high compared to the reported findings of existing studies. For instance, the prevalence of anxiety in the adult population is about 19% [67], whereas epidemiological-based studies indicated that the prevalence of insomnia is 21–25% [68]. Further, Perlis et al. [69] reported that around 30% of the population develop acute incidents of insomnia. Our findings indicated that about 2% showed symptoms of internet gaming disorder; a meta-analysis study reported that gaming disorder’s global prevalence is around 3% [70], suggesting that the gaming pattern of our study sample aligns with its global prevalence.

Our Pearson’s correlation findings indicated a significant association between insomnia and anxiety. In support of this, the association has been verified using multiple approaches [71,72,73]. A previous longitudinal report conducted in Switzerland indicated that repeated brief insomnia and continued insomnia were significantly linked to anxiety in young individuals (21–23 years). Further, the risk of insomnia reoccurrence was reasonably high [74]. Additionally, this association could lead to significant clinical consequences. For instance, functional Magnetic Resonance Imaging (fMRI) in anxiety–insomnia patients compared to healthy controls revealed a hyperactive posterior cingulate cortex and elevated network segregation. This elevation was significantly linked to the severity of insomnia [75]. In line with this, elevated functional connectivity was detected in the limbic system (amygdala) of patients diagnosed with primary insomnia [76]. Meanwhile, the amygdala is physiologically linked to emotional regulation [77]. 

A higher rate of insomnia could be attributed to multiple factors. College students, in particular, are at higher risk of acquiring insomnia, primarily due to social, academic, and professional concerns [78]. Mbous et al. reported that insomnia coexists in more than 70% of depressed students [79], indicating that having mood disorders elevates the risk of experiencing insomnia. An epidemiological study conducted on Portuguese adolescents concluded that female gender and age are substantial risk factors for acquiring insomnia [80], and a significant number of our study participants are female. In line with this, a study reported that experiencing one month of insomnia over one year was correlated inversely with age; the probability was higher in participants in their twenties and reduced compared to individuals in their thirties, then declined compared to participants in their forties. The study also indicated that insomnia is more prevalent in women [81].

A study conducted in multiple primary healthcare centers in Saudi populations from the Jeddah district concluded that severe insomnia is prevalent in younger individuals [82]. Additionally, a recent report indicated that adolescent females exhibited a higher prevalence of insomnia compared to males and younger-aged females. The report also showed that a higher prevalence of sleep reduction was linked to sleep hygiene practices, including caffeine consumption [83]. Another report on Saudi female college students indicated that coffee consumption exceeded 80% [84]. In support of this, another study on Saudi adolescents reported that 94% consumed coffee and caffeinated products [85], which could be a critical factor in the elevated rates of insomnia in Saudi college students. 

Further, the Saudi lifestyle may contribute to these findings. With its dry and hot weather, Saudi Arabia may seem dormant. But as the sun sets, a remarkable transformation occurs. The cities burst into life, offering a vibrant and energetic nightlife starkly contrasting with the daytime heat. The cities are full of glowing shopping centers. Further, Arabian culture values coffee, and coffee shops are everywhere and always crowded with people. Additionally, the majority of family gatherings, weddings, and social events are carried out during the night [86]. 

The elevated level of insomnia could be clinically relevant to the risks of developing depression [87], heart disease [88], obesity [89], and cognitive dysfunction [90]. For instance, symptoms of insomnia have been identified as a predictive risk of depression and high blood pressure. In an eight-year follow-up cohort study, it was found that individuals exhibiting four symptoms of insomnia develop both depression and elevated blood pressure [91]. In line with this, studies from the early seventies documented changes in the rapid eye movement sleep latencies in depressed patients [87,92]. Further, a report indicated that the prevalence of type 2 diabetes is more than 20% in individuals with insomnia [93]. These clinical implications of insomnia highlight the need to establish an early intervention and/or preventive measures for insomnia.

The most predominant findings within our anxiety–insomnia–gaming network structure indicated that: (1) GAD_1 and GAD_2 are interconnected at a high centrality level; (2) GAD_2 is crucial for insomnia; (3) the GAD_3 cluster was most substantial among anxiety symptoms; (4) GAD_1 and GAD_5 are essential for internet gaming disorder; (5) the centrality analysis highlighted multiple nodes; those that project the most are IGDS_7, GAD_1, and GAD_2. 

The item-level analysis found that GAD_1 and GAD_2 are interconnected at a high centrality level. Extended thinking and worrying have been linked to psychiatric [42,44] and behavioral conditions [94]. For example, a previous report indicated that GAD_1 intertwined with multiple domains of depression [44]. This connection could be established, as feeling nervous and being unable to control worrying are mirrors of persistent nervousness, which is a core symptom of anxiety [95]. 

Our findings highlighted GAD_3 as a central domain of the analysis, which could be partially driven by career entry worries [96]. Being worried too much is an essential symptom of anxiety disorder [95]. Previous research indicated that worrying too much is the central node of the depression–anxiety network in adolescents. Further, the study identified GAD_3 as a potential interventional target in adolescent individuals to mitigate the risk of the clinical consequences of depressive and anxiety symptoms [43], signifying the role of GAD_3 as a core symptom of anxiety.

Further, we found that GAD_2 “uncontrollable worrying” is crucial for insomnia. In a study examining anxiety–depression–insomnia, uncontrollable worrying was found to be a predominant node [97], which supports prior findings on the significant association between anxiety and insomnia [45,49,98]. Notably, a cross-sectional study has found that the severity index of insomnia is a predictive factor in developing psychiatric symptoms through extensive worrying and rumination [16].

Moreover, GAD_1, “feeling anxious”, and GAD_5, “restlessness”, are essential for internet gaming disorder. We found a direct link between IGDS_7 “deceiving family members regarding the amount of gaming” and GAD_1, and a connection between the IGDS_8, “use of games to escape or relieve negative moods”, and GAD_5 nodes. Both GAD_1 and GAD_5 are essential for internet gaming disorder and are supported by the literature as key symptoms of anxiety that are essential for other mental conditions [42]. Additionally, GAD_5 is a core psychomotor symptom of anxiety [95]. A systematic review demonstrated that behavioral addiction, including gaming and gambling, was driven by poor emotional regulation. These behavioral addictions were mediated by seeking an escape from negative emotions [99]. Further, in adolescents, emotional regulation is directly linked to the time spent on gaming [100], even though, in our sample, the risk of exhibiting internet gaming disorder is minimal. This finding could be crucial for understanding the factors correlated with gaming addiction and considering that video gamers promote elevated levels of stress, loneliness, anxiety, depression, and alcohol use disorder [101]. 

Most of the existing literature utilizes network analysis on the general population using survey-based studies, which highlights the risk of developing a psychiatric disorder [42,43,46,48]. It has also been used for randomized controlled trial studies. For example, a previous report examined the impact of cognitive behavioral therapy on symptoms of insomnia and depression using network analysis. The study findings indicated a sequential improvement in insomnia symptoms driven by cognitive behavioral therapy in depressed individuals. These improvements started in the first week of treatment, with early morning awakening symptoms depicted by the Insomnia Severity Index scale, and the improvement in individuals’ dissatisfaction symptoms was reached by the fourth week of treatment [102]. Another translational aspect of employing network analysis in psychopathology is examining behavioral and biological connectivity. This clinical analysis has been utilized in a study investigating the connectivity of peripartum depression and the biological markers of stress and reproduction. Item-level analysis indicated that dislike—estriol, fear—a corticotropin-releasing hormone, cry—cortisol, and loneliness—cortisol were interconnected in depressed pregnant Latina women [103]. Therefore, it is for future studies to translate these findings by establishing a biochemical association, such as the cortisol serum level, or by interview-based psychological assessment.

It is worth mentioning that previous studies examined the association between insomnia and anxiety. However, this is the first report to examine insomnia and anxiety in an item-based analysis with respect to gaming addiction. This is also the first report to examine this association in an internship sample population. One interesting finding was the lesser prevalence of gaming addiction in our sample. Most importantly, our findings indicated that “feeling anxious”, “restlessness”, and “uncontrollable worrying” are central symptoms in this association. Therefore, a key implication is needed for studies translating this knowledge via behavioral strategies to target these disturbances [104,105,106]. Another intervention method is establishing a campus-based counseling program. A support group consisting of experts and peer students would facilitate the student’s stress coping and understanding of their feelings [107,108,109].

Our findings of the anxiety–insomnia–gaming network align with contemporary studies that have identified Dysregulation of Mood, Energy, and Social Rhythms Syndrome (DYMERS) [110,111]. The DYMER syndrome is clinically significant in the progression of health conditions. For example, exposure to stress represents a risk factor for the bipolar spectrum [112]. Further, DYMER is manifested by the dysregulation of behavioral, social, and biological rhythms such as sleep, stress, diet, eating habits, and sociability [110,111,113]. These studies highlighted the research gaps in social and behavioral rhythmic domain dysregulation. 

**Limitations of the study:** While the study at hand is novel and significant, with network analysis providing a comprehensive profiling of the study elements, it is crucial to acknowledge the presence of certain limitations. The study design, for instance, is cross-sectional. Although commonly used, this design limits the ability to establish a causal interface between the study variables. Secondly, the sample was collected via convenient snowball sampling. Random sampling could overcome sample and study bias representation. Additionally, it is important to note that recall bias is a potential factor that cannot be entirely ruled out. The influence of self and social desirability on participant responses could impact the validity of the study’s findings.

Furthermore, anxiety and insomnia are highly comorbid and affected by physiological and psychological health conditions, and we did not include any clinical variables within the study. Finally, network analysis is promising, and emerging in the context of psychiatric and psychological studies. Yet, studies examining the accuracy and stability of this analysis framework are sparse [114]. Future studies should consider these limitations using a prospective longitudinal cohort within a larger sample size. 

## 5. Conclusions

We found high rates of anxiety and insomnia, and an association between selected symptoms of anxiety and insomnia. The Saudi lifestyle may contribute to these findings. Low gaming addiction rates could be attributed to a lack of entertainment time and increased risk awareness. Deliberation anxiety–insomnia–gaming network domains are beneficial in identifying proper psychological and functional interventions. Given these findings, an awareness of anxiety and insomnia risks should be emphasized.

## Figures and Tables

**Table 1:** Demographic characteristics of students (*N* = 263).

Variable	Category	*n*/*M*	%/*SD*
Age	Range 18–30	22.83	1.37
Sex	Male	85	32.319
	Female	178	67.681
Relationship status	Single	252	95.817
	Married	11	4.183
Number of family members	Range 1–14	6.41	2.35
Internship program—college -	Applied medical sciences	120	45.63
	Medicine	10	3.8
	Dentistry	18	6.84
	Nursing	41	15.59
	Pharmacy	55	20.91
	Humanities	2	0.76

**Table 2:** Frequencies of particular categories of internet gaming disorder, anxiety, and insomnia symptoms.

Variable	Categories	*n*	%
Internet gaming disorder	Non-disordered gamers	257	97.72
	Disordered gamers	6	2.28
Generalized anxiety disorder	No symptoms	92	34.98
	Mild symptoms	95	36.12
	Moderate symptoms	54	20.53
	Severe symptoms	22	8.37
Insomnia	No insomnia	104	39.54
	Insomnia	159	60.46

Note. Disordered gamers can be identified if they respond “Very often” to at least five of the nine items on the IGDS9-SF. Insomnia is considered with a score of six or more in the AIS.

**Table 3:** Descriptive statistics (*N* = 263).

Variable	Range	*M*	*SD*	Skewness	Kurtosis	Cronbach’s α
Internet gaming disorder	9–41	18.53	7.84	0.64	–0.49	0.89
Anxiety	0–21	7.22	5.06	0.56	–0.32	0.89
Insomnia	0–23	7.06	4.52	0.52	–0.03	0.83

## Data Availability

Data are available upon reasonable request.

## Supplementary Materials

The following supporting information can be downloaded at: https://www.mdpi.com/article/10.3390/jcm13144054/s1, Table S1: Centrality measures per variable.
