# Supplementary material for: Examining Anxiety and Insomnia in Internship Students and Their Association with Internet Gaming Disorder
Source: J Clin Med. 2024 Jul 11;13(14):4054. doi: 10.3390/jcm13144054 (PMC11278388; doi:10.3390/jcm13144054)
Supplement: Supplementary file 1 [file jcm-13-04054-s001.zip › jcm-3047969-supplementary.pdf]

Supplementary Materials:

Examining Anxiety and Insomnia in Internship Students and Their Association with Internet Gaming Disorder

Tahani K. Alshammari, Aleksandra M. Rogowska, Anan M. Alobaid, Noor W. Alharthi, Awatif B. Albaker, and Musaad A. Alshammari

Supplementary Table S1. Centrality measures per variable

	Network analysis			
Item	Betweenness	Closeness	Strength	Expected influence
IGDS_1	-1.23	-0.86	-0.24	-0.99
IGDS_2	-0.55	-0.01	0.70	1.09
IGDS_3	1.04	0.18	1.38	1.15
IGDS_4	-0.78	-1.27	-0.98	-0.68
IGDS_5	-0.40	-0.45	0.57	0.99
IGDS_6	-0.93	-1.12	0.15	0.54
IGDS_7	2.48	1.80	1.10	-0.43
IGDS_8	0.59	1.00	-1.10	-0.82
IGDS_9	0.21	-0.16	-0.10	-0.37
GAD_1	1.50	1.91	0.75	0.31
GAD_2	1.50	1.34	1.77	2.30
GAD_3	-0.55	0.73	0.57	0.36
GAD_4	0.21	0.65	-0.22	-0.28
GAD_5	0.44	0.79	0.71	0.83
GAD_6	-1.16	-0.89	-0.85	-0.54
GAD_7	-1.16	-0.63	0.02	-0.07
AIS_1	-1.16	-1.23	-1.75	-1.52
AIS_2	-0.02	-1.05	-0.25	-0.20
AIS_3	-1.00	-1.80	-1.31	-1.05
AIS_4	0.21	0.48	0.17	0.56
AIS_5	1.27	0.74	0.62	-0.40
AIS_6	-0.47	0.09	0.69	1.13
AIS_7	0.13	-0.01	0.00	0.33
AIS_8	-0.17	-0.25	-2.40	-2.22

Note. IGDS = Internet Gaming Disorder Scale, GAD = Generalized Anxiety Disorder; AIS = Athens Insomnia Scale. The indices are shown as standardized z-scores.
